# Patient Perceptions of the Impact of Treatment (Surgery and Radiotherapy) for Soft Tissue Sarcoma

**DOI:** 10.1155/2019/9581781

**Published:** 2019-01-23

**Authors:** Lauren Hewitt, Rachael Powell, Kaan Zenginer, Catherine Coyle, Helen Murray, Lisa Cooper, Jonathan Gregory

**Affiliations:** ^1^School of Health Sciences & Manchester Centre for Health Psychology, University of Manchester, Manchester, UK; ^2^The Christie NHS Foundation Trust, Manchester, UK; ^3^Manchester University NHS Foundation Trust, Manchester, UK; ^4^The Royal Orthopaedic Hospital NHS Foundation Trust, Birmingham, UK; ^5^Molecular and Clinical Cancer Sciences, School of Medical Sciences, University of Manchester, Manchester, UK

## Abstract

**Background and Objectives:**

Treatment for soft tissue sarcoma (STS) is challenging for patients. This study aimed to gain an in-depth understanding of patients' experiences of STS treatment, including whether the sequence of treatment (preoperative or postoperative radiotherapy) influences patient perceptions.

**Methods:**

Face-to-face semi-structured interviews were conducted with nineteen patients who had been treated for STS with surgery and radiotherapy between 2011 and 2016. Topics discussed included perceptions of treatment, social support, and coping mechanisms. Qualitative, inductive, thematic analysis was conducted and structured using the Framework approach.

**Results:**

Treatment sequence itself did not appear to cause concern, but uncertainty regarding treatment and side effects could negatively impact participants. Social relationships and individual coping strategies influenced participants' experiences of treatment.

**Conclusions:**

Participants' perceptions of the treatment process varied; the experience was highly individual. It is important to ensure individual psychosocial and information needs are met. In particular, the removal of uncertainty regarding treatment is important in supporting patients undergoing treatment for soft tissue sarcoma.

## 1. Introduction

Sarcomas are rare, aggressive tumours that represent 1% of cancerous growths in adults [1]. Soft tissue sarcomas (STSs) develop in supporting or connective tissues, such as muscles, nerves, and fibrous tissues [2]. Although STS can affect any part of the body, they most commonly occur in the extremities [3]. They are often associated with physical or psychosocial difficulties and a high risk of recurrence [4, 5].

Treatment for STS is managed by multidisciplinary teams [6]. A combination of surgery and radiotherapy is the standard treatment for patients with extremity STS [1], but treatment plans vary depending on a patient's comorbidities and the size, origin, and grade of the tumour [7]. Treatment can lead to functional disabilities and side effects of radiotherapy [8, 9].

Patients may now receive preoperative, rather than postoperative, radiotherapy [5, 6]. Preoperative radiotherapy can last 5 weeks, after which there is a deliberate wait of 4–6 weeks before surgery to remove the tumour [5]. Anecdotal reports by clinicians suggest that patients are concerned about receiving preoperative radiotherapy due to the extended wait to remove the tumour, but there is a lack of research investigating the issue.

Insight into patients' perceptions of treatment for STS primarily comes from quantitative research. Patients with STS can experience negative effects throughout treatment, including clinically significant levels of depression and anxiety [4, 10], changes in appearance and mobility, and feelings of pain and fatigue [11–13]. These experiences may be a consequence of the intensive treatment regimens associated with sarcoma, which can disrupt patients' lifestyles and mental and physical abilities [14].

One qualitative study of ten participants on the treatment-related experiences of patients with STS found that patients had concerns such as fear of changing life roles, loss of life, and impact on employment [15]. Since this study was published in 1999, treatment has evolved, particularly in terms of treatment order [5, 6], so patient experiences may also have changed.

A recent systematic review on patient experience and quality of life following STS diagnosis found that patients experienced a range of adverse psychological and physical effects [16]. This review specifically highlighted the need for up-to-date, qualitative research into the area, as quantitative research methods do not permit in-depth exploration of perceptions; thus, studies may fail to represent the full impact of treatment on patients [10]. It is important to have a detailed and nuanced understanding of patient experiences of STS treatment so that health professionals can effectively meet the needs of patients before, during, and after treatment, providing appropriate information, reassurance, and support.

The present study aimed to understand whether treatment sequencing (preoperative or postoperative radiotherapy) influenced patient perceptions. It aimed to gain a deeper understanding of patients' perceptions of treatment for STS, identify concerns throughout treatment, and consider what patients found helpful. Qualitative methodology was used because it facilitates a rich, detailed exploration of individual patient experiences and can uncover meanings and understandings of experiences.

## 2. Materials and Methods

### 2.1. Design

Single, semi-structured face-to-face interviews were conducted with individuals who had been treated for STS.

### 2.2. Participants

Participants were identified by members of a sarcoma multidisciplinary team from records of patients treated for STS at a hospital in the North West of England. Inclusion criteria were as follows: diagnosed with STS within the last five years (between 2011 and 2016), not currently receiving radiotherapy or chemotherapy treatment, residing within the local area, good understanding of the English language, and older than eighteen years. Patients judged by the health professionals to have a particularly poor prognosis or for whom psychological well-being was a concern were not approached.

### 2.3. Procedure

Health professionals informed eligible participants about the study during routine clinical appointments. Patients were given an information pack containing an invitation, information sheet, response sheet, and prepaid envelope. Patients without an upcoming clinical appointment were posted an information pack. Researchers contacted participants who returned a response sheet to confirm eligibility, answered any questions the participants had, and arranged an interview.

Literature on patients' experiences throughout cancer treatment guided the design of the interview schedule [15, 17, 18]. Interview topics included the following: order of treatment, experience of the treatment process, social support, and coping mechanisms. The semi-structured format allowed participants to speak freely and to influence the depth and content of the interview.

Interviews took place at participants' homes or in a room on the university campus and were conducted by two postgraduate researchers between June and August 2016. Informed consent was obtained from all individual participants included in the study. Participants chose a pseudonym to ensure anonymity. Interviews were audio-recorded and transcribed verbatim.

Thematic analysis was conducted with the goal of gaining a meaningful understanding of participants' experiences and perceptions [19]. Themes were derived inductively (without reference to a preexisting model) from the interview data [19]. The analysis was conducted manually and structured using the Framework approach, a systematic and transparent process which facilitates comparison and contrasting of data both across and within cases [20]. Five steps were followed: Familiarisation: interviews were extensively read and reread. Coding: substantive codes relating to interesting features of the data were noted [19]. Codes were refined and grouped together if conceptually related to develop a working analytical framework of thematic categories. Indexing: the refined codes were attached to interview transcript excerpts. Charting: the data from each participant and each refined code were summarised in a framework matrix [20]. Mapping and interpretation: themes and subthemes were developed, drawing connections within and between each code. Reliability was enhanced through an iterative process, during which two authors discussed and refined codes and themes [21].

## 3. Results

Fifty information packs were distributed to patients. Nineteen responded (38% response rate) and continued to interview. Participants were aged between 29 and 84 years (median 65 years); eight were female and eleven male. Nine participants received preoperative radiotherapy and ten received radiotherapy postoperatively. Six participants were currently working, ten were retired, and three were not working due to ill health. Participants reported eleven different histological subtypes of STS diagnosis; five did not recall their STS type. Time since diagnosis ranged from 7 to 48 months (median 22) and time since treatment ended ranged from 2.5 to 48 months (median 18). Interviews ranged from 36 to 76 minutes (median 48).

### 3.1. Themes

Three main themes were identified: care process, coping with treatment, and social relations throughout treatment. Participant quotes are presented with pseudonyms.

#### 3.1.1. Care Process

*(1) Experience of Treatment*. Some participants wanted their tumour removed immediately: “*just get it out, it's cancer, just get it out*” (Liz) and expressed confusion and anxiety over their treatment plans: “*I was scared, because I don't know what radiotherapy was, or what effect it was going to have*” (Liz). Most participants said that the initial concern or uncertainty upon hearing their treatment plan was reduced when information about STS was provided and the reasons behind their treatment plans were explained by health professionals:

*Once you know that (understanding of treatment plan), you can get on with your life because it's, that's what it is isn't it.* (Jonathon)

Many participants said they accepted their treatment plan: “*Fine. Yeah, it's just; it's just what you have to do*” (Heather). Participants acknowledged that they would need to receive some form of treatment for STS and expressed high levels of trust in the health professionals and decisions made by multidisciplinary teams. This applied to participants receiving preoperative or postoperative radiotherapy:

*He said having the radiotherapy first was to stop it spreading? Or anything round it or something to kill off? … Um yeah, I was quite comfortable with what they were telling me.* (Mary)

One participant who received radiotherapy prior to surgery voiced concerns and frustration over delays that the order of treatment appeared to have on treatment commencing, with delays in radiotherapy contributing to worry:

*I've already said “ok I'll accept radiotherapy,” well, I didn't think we'd do radiotherapy first, but now we've wasted another three weeks before the radiotherapy starts. If it was surgery, you could have taken it out almost three weeks ago before I started radiotherapy.* (Simon)

Despite initial concerns, Simon had accepted his treatment plan as he felt trust in the multidisciplinary team, but delays in accessing radiotherapy seemed to lead to anger, frustration, and erosion of his trust in the care team.

Some participants reported being given the option of treatment order and/or continuing treatment. Those who were given the option of pursuing treatment reported choosing the perceived long-term health benefits by continuing as opposed to avoiding treatment, despite potential physical or psychological side effects:

*There's that embarrassment factor you know thinking “I don't know if I can do it. I don't know if I can have the radiotherapy” But in the end, I said “Yeah ok, probably the best thing to do.”* (Luke)

Being given treatment options affected people in different ways. Some seemed pleased, while others expressed confusion that led them to misunderstanding that they may not receive treatment and questioned why this might be:

*He (surgeon) said “Well, there's the operation or we could possibly leave it.” That could've been quite wrong because there was obviously a problem, which surprised me. He possibly thought correctly, that because the other one wasn't a problem, this was probably, and because of my age, he'd leave it.* (Ronnie)

Some participants found the apparent inconsistency between waits for appointments and urgency to be treated frustrating and confusing, as even the shortest wait can feel arduous when time is considered to be an important factor with a disease:

*On the one hand, you're told you need something done quickly, and obviously you feel that you want something done quickly and yet, well, it's not possible to do it because of the various delays.* (Greg)

These findings highlight the importance of patient understanding throughout the treatment process and the difficulty of explaining treatment to patients in complex cancers such as STS where care is highly individualised. Ensuring that patients fully understand reasons behind treatment decisions, options, and possible delays may reassure patients and maintain their trust in the health-care team.

*(2) Posttreatment Perceptions*. Often, the reality of treatment was found to be less severe than anticipated:

*We didn't have to worry about anything, did we? Not the treatment, not anything, it was a breeze if you want. It was so easy.* (Heather)

Participants typically spoke of feeling grateful to have been treated and were in admiration of the health professionals who delivered their care:

*So, the whole sort of attitude and the staff training there, whoever is responsible for the training there really deserves a star because they really are excellent.* (Greg)

These feelings were consistent across the sample and were often reinforced when the worst possible outlook was considered, for example, loss of limb or life:

*They managed to save my leg and until then I hadn't really appreciated that I might have lost a leg, yeah so whatever the pain was and the after effects and so on, I was just very thankful that they had managed to save it.* (Helen)

Some participants found the fear of recurrence difficult, particularly prior to follow-up medical appointments when anticipatory fear was reported to increase. These feelings were said to subside after receiving scan results at the appointment, but would gradually return with every additional correspondence and appointment. This suggests that STSs may have a long-term emotional impact:

*I tend to live my life in three month chunks at the moment, with these three-monthly check-ups, you know, you get a bit sort of, you, feel a bit on edge, at the date of the appointment the chest x-ray, come along.* (Simon)

However, people also expressed the belief that the negative emotions they felt throughout treatment were likely to subside as time passes and STS becomes less of an immediate threat:

*It doesn't worry me like it used to because I've realised it's 3 years and I'm still here. And I can do what I did before but it does take a long time to realise that, it took me a long time.* (Anna)

#### 3.1.2. Coping with Treatment

*(1) Coping Strategies*. Participants discussed a range of coping strategies used throughout treatment to manage negative emotions or experiences, including side effects. Remaining optimistic throughout treatment was said to be important; some participants suggested that maintaining a positive attitude made the situation seem more bearable for themselves and others:

*I made a really positive decision, especially for all the people round me, that I didn't want to be this poorly person that's got cancer.* (Anna)

Similarly, some participants said that they focused on a specific goal or point in the future to help them to stay motivated throughout treatment and remained hopeful that they would reach this point:

*I always thought about I'm going to see my mum, within weeks. That's kept me running. Otherwise very hard, psychologically very hard.* (Ahmed)

Participants said that by thinking about treatment and their current situation dispassionately, they were more able to justify and understand difficult emotions or events. Some participants appeared to do this by weighing up the pros and cons of treatment, using downward social comparisons (comparison of themselves to someone in a worse position) and not letting emotion dictate current feelings:

*I just kind of thought “There are lot more things I could have at my age you know. I could have a degenerative neurological condition you know. I could have a cancer of the pancreas or something that's kind of going to do me in very quickly” and it all just seemed very rational.* (Sarah)

Increasing age also appeared to be a factor in how some individuals thought about treatment outcomes. Older participants reflected on their life up to the present time and thought of themselves as fortunate to have already experienced a fulfilled life:

*When you reach a certain age, there's no point thinking about “oh dear I shouldn't get this.” It's luck of the draw, so erm, so I feel fine. If my end is near, well I've had a good life, so who knows?* (Sophie)

*(2) Approaches to Information*. All participants had wanted basic information about their diagnosis, treatment plans, and potential outcomes. Despite some participants having no prior knowledge of STS, many said they received sufficient information from health professionals. Others appeared to seek more information and utilised online resources such as Google *“he said it's a soft tissue cancer, sarcoma. I'm like, pfff, no idea. Googled that one when I got home”* (Joe). These participants felt that a better understanding of treatment helped improve their emotional response to treatment, as they knew what to expect:

*Because people don't know enough information I don't think and that's, that's one of the big down sides because they don't know stuff they, they think the worst don't they and people do that projection thinking ooh gosh I wonder if I wonder if and drive people nuts, including me at times … so I asked tons of questions all of the time, want to know everything.* (Luke)

By contrast, some appeared to have the perception that “*some things are best left unknown*” (Helen)*;* that having more information may increase worry. Information-seeking behaviour varied across an individual's treatment process and with individual preferences.

Some patients, as well as expressing a desire for less information, used distraction as a way of managing the negative feelings that sometimes accompanied treatment. Continuing to work, taking part in hobbies, and socialising were found to be helpful as participants focused on something other than STS. This seemed to increase feelings of self-worth:

*It helped, you know, that I still had a use you know I could still do something, I wasn't helpless, I wasn't a victim sort of thing, you felt that you were I don't know, in the world of the living.* (Helen)

It seemed that each participant displayed emotions and used coping strategies unique to them, illustrating that there is no set way of coping with treatment. Participants themselves acknowledged this variation and the uniqueness of each experience:

*It's going to be a personal journey, there's going to be the generic parts like the radiotherapy. But how you actually, how you as a person react to all parts of the journey is totally different.* (Liz)

#### 3.1.3. Social Relations throughout Treatment

Participants derived psychological and physical support from a network of family, friends, community members, and social support groups. Most participants specifically reported a “significant other” (spouse, parent, or close friend) as important to helping them cope throughout treatment:

*So, without my mum I don't know what would have happened … basically anything that I needed she helped with.* (Derek)

A number of participants spoke about the isolation or sense of being alone that accompanies being treated for STS, even when emotions and experiences were shared with others:

*It's very lonely because even when you've got all your family around at the end of the day it just comes down to you, doesn't it?* (Anna)

Although all participants were aware of additional sources of support, such as Macmillan (a UK charity that provides support to people with cancer), the extent to which these services were used appeared to be inversely related to how much support was received from close family and friends:

*I was offered counselling, but I didn't feel I needed it, because I just felt so loved and looked after by all my friends and my family.* (Anna)

Individuals who accessed support groups reported doing so as they wanted someone to relate to. This seemed to be beneficial for participants as it enabled them to feel understood by someone who had experienced a similar situation:

*I think because they (patient support groups), they sort of understood how I was feeling, whereas nobody else really did, well they can't can they? My family members didn't really understand what was going on in my head.* (Lola)

Whilst social support could be beneficial, interaction with others could also be perceived to have a detrimental impact. This was notable if participants had a tempestuous relationship with others, or when participants felt an expectation to act, feel, or behave in a certain way, making it difficult for some patients to seek support. Some participants reported altering their behaviours to comply with these expectations:

*I think they just sort of expect me to get on with it, and so you do. They don't want to listen to me moaning, so I don't.* (Helen)

The interplay between how treatment affects the patient and the demands it imposes on the caregiver/nonpatient was also discussed:

*She (mum) would say “well this does affect me, it does upset me” (…) I was like just suck it up a bit, you know coz you're getting upset now you're putting that on me, so I've got to deal with all this and how I feel and also now you know, we've dealt with you! Yeah that was frustrating.* (Derek)

It seems that the perceived need to accommodate or consider other people's feelings alongside coping with the impact of STS on themselves could be challenging.

Interviewees also expressed concerns over the impact their treatment and outcome may have on their children and others which, in turn, worsened their own anxiety:

*I don't know how someone could not be anxious; maybe because I had two kids and this awful family situation that's what made it a lot worse for me.* (Hugo)

However, others seemed to find the desire to protect other people beneficial; appearing optimistic around others seemed to help some participants manage their own emotions:

*Because my mum was there I didn't get upset, whereas if my mum hadn't have been there I think my reaction would have been a lot worse, but I felt as though I had to hold it together a bit, for her mainly.* (Lola)

## 4. Discussion

This study found that participants identified various concerns throughout treatment, such as a lack of understanding of what STS was and apprehension about treatment plans. Participants used a range of strategies to help them cope throughout treatment, including information-seeking behaviour, and had a general need for social support; the themes highlight the experience and aftermath of treatment for STS as psychologically challenging. These findings are in line with existing research into patient perceptions of sarcoma treatment [9, 15, 16].

In the present study, most participants did not express concerns over the order of treatment once the reasons behind the proposed treatment had been explained. Thus, evolutions in medical treatment do not of themselves seem to be impacting patient experience. Instead, concern arose from uncertainty surrounding treatment plans, perceived treatment delays, and possible side effects. Understanding of treatment for STS may come from verbal information provided by health professionals or from educational materials provided at diagnosis. Having readily available information provides patients with the option of reading further and is important for positive health outcomes, such as alleviating stress, improving confidence in treatment, and reducing levels of anxiety [22]. However, given the different information-seeking behaviours and the complex nature of STS treatment, it is unlikely that a single approach to information will suit all patients. Health-care professionals should be prepared to supplement standard information with personalised advice to suit patients' situations and information preferences throughout the treatment and follow-up journey to ensure that uncertainty regarding treatment and side effects is minimised.

The findings show that participants can perceive different aspects of treatment to be psychologically challenging, for example, anxiety in the lead-up to appointments or the potential impact of their treatment on others. This is consistent with research which has found that treatment for STS can be the trigger of stress and negative emotions [4]. It also demonstrates that individuals acknowledge and appraise stressors and aspects of treatment differently. The findings highlight how participants attempt to overcome stressors using coping strategies such as downward social comparisons and distraction. It has been found that people frequently, and effectively, use downward social comparisons and distraction styles of coping throughout treatment for cancer to regulate their emotions and enable them to better adjust to their situation [23, 24]. Supporting patients to use different coping strategies could help them manage difficult emotions throughout treatment.

Participants expressed a need for social support during their treatment. Talking to others during the treatment process seemed to provide patients with someone to relate to, reassurance, and an increased sense of self-worth—implying that treatment for STS could be perceived as being more bearable if social support networks are utilised. Emotional support has been associated with positive outcomes for cancer survivors, including decreased social disruption and improved general mental health [25–28]. Research has also found that patients who feel they do not have enough good quality support may turn to external sources, such as support groups [25]. This was reflected in the current study as patients reported using groups such as Macmillan if they felt close support was not sufficient. Some participants seemed to feel that social relations caused difficulties at times during treatment, as they perceived that they had to provide support for others, or were concerned for others' welfare. Factors such as a patient's age or gender can impact on social support needs [15, 26], for example, younger adult patients often experience unmet childcare needs [29]. Given that STS is more commonly found in younger adults [30], it is possible that the emotional support needs of STS patients will differ to people with other cancers as younger adults might have greater parental engagement or caregiving responsibilities [29]. The continued incorporation of social support needs and preferences in STS treatment plans will allow the appropriate support services to be provided.

This study's sample included participants with a wide age range (29–84 years), and it seems that life stage may have some influence over people's experiences of soft tissue sarcoma. Some older adults reflected on their illness in terms of having already lived a fulfilled life, whereas some of the younger participants had concerns as to how the treatment and outcome might affect their children and parents. However, the present study did not set out to examine people's experiences by age group, so further research which purposively samples people in different age groups could usefully provide further evidence regarding these initial findings.

The aim of qualitative research is not to generalise findings across an entire population but to provide a more detailed, nuanced understanding of experiences than would be possible with a larger sample size. The final interviews yielded no new ideas, suggesting theoretical saturation had occurred and the sample size of 19 was appropriate [19]. The participants in this research were those individuals who responded positively to study information and opted in to the study, and it is possible that participants had different experiences with STS treatment to those who chose not to take part. However, the present sample was diverse in terms of perceptions of treatment, age, gender, type of STS, and treatment order. The interviewers had no clinical relationship with the participants. As a result, some contextual information may have been missed, but it also meant the participants could talk freely about their experiences of treatment without worrying about their disclosures having any potential impact on their care. Patients who were actively undergoing treatment or deemed to have a poor prognosis were not interviewed as the narratives of the different patient groups were likely to differ significantly, both in terms of optimism and reflection over the treatment process. Future research should include these populations to understand how treatment plans are perceived at the time of treatment or when expectation of survival may be lower.

## 5. Conclusion

As one of the few qualitative studies to research patient perceptions of STS treatment, this research provides a much needed insight into the potential impact of treatment on patients. It was found that while the order of treatment (radiotherapy and surgery) did not itself seem to cause concern, uncertainty regarding treatment and side effects could have a negative impact. A patient's experience of treatment can be affected by their understanding of treatment plans, their social support experiences, and their use of coping strategies. Experiences of each of these factors were diverse. Thus, it is important that health-care professionals have time made available throughout the treatment process to be able to identify and meet the individual psychosocial and information needs and preferences of patients.

## Data Availability

The interview transcript data used to support the findings of this study have not been made available in order to protect patient privacy. Participants have consented to data being accessible only by the university research team members or by authorized individuals from the university, from regulatory authorities, or from the NHS trust for monitoring and auditing purposes. This approach was approved by the North West–Greater Manchester Research Ethics Committee.
